# Access-related infections in two haemodialysis units: results of a nine-year intervention and surveillance program

**DOI:** 10.1186/s13756-019-0557-8

**Published:** 2019-06-18

**Authors:** Ittamar Gork, Ilana Gross, Matan J. Cohen, Carmela Schwartz, Allon E. Moses, Michal Dranitzki Elhalel, Shmuel Benenson

**Affiliations:** 1 Department of Nephrology, Hadassah-Hebrew University Medical Center, Jerusalem, Israel; 2 Department of Clinical Microbiology and Infectious Diseases, Hadassah – Hebrew University Medical Center, POB 12000, 9112001 Jerusalem, Israel

**Keywords:** Access-related infections, Haemodialysis, Intervention, Surveillance, Checklist

## Abstract

**Background:**

Access-related infections are a major cause of morbidity and mortality in haemodialysis patients. Our goal was to decrease the rate of these infections by implementing an intervention and surveillance program.

**Methods:**

This intervention took place in two haemodialysis units (Units A and B) and was a joint effort by the haemodialysis staff and the unit for infection prevention and control. It included reviewing the work methods and work space, observations on compliance with standard precautions and handling of the vascular access, creating a checklist and a designated kit for handling the vascular access and prospective surveillance of access-related infections.

**Results:**

During a nine-year period, the haemodialysis units A and B treated 4471 and 7547 patients (mean number of patients per year: 497 (range 435–556) and 839 (range 777–1055), respectively). For most patients, the procedure was done through an arteriovenous fistula (66.7%, range 50.3–81.5%). The access-related infection rate decreased significantly in both haemodialysis units: from 3 to 0.9% (trend: *p* < 0.05, linear regression: *p* < 0.001) in Unit A and from 0.9 to 0.2% (trend: p < 0.05, linear regression: *p* = 0.01) in Unit B.

**Conclusions:**

An intervention which included introduction of a checklist and designated kit, together with ongoing surveillance and feedback, resulted in a significant decrease in the access-related infection rates in both haemodialysis units.

## Background

Infection is the second leading cause of death in haemodialysis patients [1]. Bloodstream infections are a common cause for morbidity and mortality in these patients, many associated with the presence of a vascular access. The incidence of access-related bacteremia is estimated at 0.9–2.0 episodes per one catheter year [2]. The type of vascular access is a critical risk factor for bacteremia. The risk for an access-related bacteremia with a tunnelled catheter is ten times higher than with an arteriovenous (AV) graft and twenty times higher than with an AV fistula [3]. Surveillance is known as an important factor in preventing access-related bacteremia in haemodialysis patients [4]. In 2009, the Centers for Disease Control and Prevention (CDC) published a list of nine core intervention components for dialysis bloodstream infection prevention including hand hygiene and vascular access observations, staff and patient education, disinfection techniques and surveillance [5]. We present the first 9 years of our intervention and surveillance program in two haemodialysis units in a tertiary care center in order to decrease the access-related infection rates.

## Methods

### Setting

Our intervention and surveillance program was carried out at the Hadassah-Hebrew University Medical Center, Jerusalem, Israel, an 800 bed urban academic tertiary referral center. It included two haemodialysis units under supervision of the nephrology department of Hadassah: the in-hospital 15-station unit of Hadassah Ein-Kerem, treating inpatients and outpatients (Unit A), and the 17-station outpatient only unit (Unit B). Only outpatients were included in our study. The patient population of Unit A is comprised of patients with a high degree of comorbidities, including oncologic and hematologic patients. Unit B treats more ambulatory patients with less comorbidities (younger and with a better functional status). On December 2015, Unit B was transferred to a new facility within the hospital, increasing its capacity for haemodialysis patients from 15 to 18 stations and allowing it to handle more complex patients.

### Data collection

Since January 1st 2009, a nursing staff member of each unit collected data prospectively about each of the following events: hospitalization, antibiotic treatment, positive blood culture, vascular exit site infection or catheter exchange due to infection. For each event, data was collected about the type of event, type of vascular access, reason for hospitalization (if occurred), blood culture results and vascular access culture results. A census form documenting the number of outpatients treated in each unit and their type of vascular access (AV graft, AV fistula or tunnelled catheter) was completed in the first two work days of each month.

### Identification of access-related infection

At the end of each month, each event was assessed by the program’s infectious disease specialist (SB) together with the program’s infection control nurse (IG) to determine the presence or absence of access-related infection. The infectious disease specialist and infection control nurse were not involved in routine patient care. They reviewed all clinical, imaging, laboratory and microbiology results and based their evaluation on the CDC criteria for nosocomial infections [6]. Briefly, vascular access exit site infection was defined when pus, redness or swelling of the access site was present without a bloodstream infection that can be related to the vascular access. Bloodstream infection was defined as related to the vascular access when no other source for the bloodstream infection was found and considered secondary when a primary source of infection was present. Both exit site infection and access-related bloodstream infection, were considered as an access-related infection. In the presence of infection, we manage the patient and the catheter according to the Infectious Diseases Society of America (IDSA) clinical practice guidelines for the diagnosis and management of intravascular catheter-related infection [7].

### Intervention

At the beginning of the intervention and surveillance program, infection control staff (nursing and medical) reviewed the working methods in both haemodialysis units. This review included multiple observations examining compliance with standard precautions, handling the vascular access and evaluation of the workspace. Recommendations and guidelines for improvement were issued accordingly. These included: 1) Creating a checklist for handling of the vascular access through all the steps during the haemodialysis session (Table 1). 2) Designing a structured kit, based on the checklist, containing all necessary equipment and composed of three components: a) dressing change; b) connecting the vascular access and c) disconnecting the vascular access. The kit was arranged according to the flow of procedures during the haemodialysis session (Table 2). 3) Emphasizing the advantage in performing shunt operations to minimize the number of patients doing dialysis through a tunnelled central catheter. 4) Establishing staff and patient education protocols. 5) Implementation of a monitoring and reporting system for infectious episodes. 6) Presenting the data to haemodialysis units nursing and medical staff quarterly and annually and discussing in periodical meetings. During the implementation of the intervention and periodically thereafter, the infection prevention team (nursing and medical) reviewed the working methods in both haemodialysis units. This included observations on the process of handling the vascular access.

**Table 1:** Checklist for handling vascular haemodialysis access

Dressing change	
Perform hand hygiene^*^	Yes/No
Wear a surgical mask (nurse and patient)	Yes/No
Wear non-sterile disposable gloves	Yes/No
Remove the dressing from the catheter without touching adjacent skin	Yes/No
Remove gloves and perform hand hygiene	Yes/No
Open the dressing change kit	Yes/No
Pour 2% chlorhexidine solution into a bowl	Yes/No
Perform hand hygiene and put on sterile gloves	Yes/No
Place three swabs in the chlorhexidine bowl	Yes/No
Disinfect the exit site thrice, once with each swab; wait for the area to dry	Yes/No
Wrap the catheter hubs with a sterile gauze	Yes/No
Cover the exit site with a sterile dressing	Yes/No
Remove gloves and perform hand hygiene	Yes/No
Connecting the patient to the haemodialysis machine (immediately after dressing change)	
Open the kit for connecting the patient	Yes/No
Pour 2% chlorhexidine solution into a bowl	Yes/No
Wear sterile gloves	Yes/No
Put gauze pads in the chlorhexidine bowl	Yes/No
Place a sterile towel beneath the catheter	Yes/No
Clean the catheter hubs with the gauzes	Yes/No
Draw dead space from each lumen	Yes/No
Connect the haemodialysis tubing	Yes/No
Remove gloves and perform hand hygiene	Yes/No
Disconnecting the patient from the haemodialysis machine	
Perform hand hygiene and put on non-sterile gloves	Yes/No
Wear a surgical mask (nurse and patient)	Yes/No
Return blood from the machine	Yes/No
Measure blood pressure	Yes/No
Remove gloves and perform hand hygiene	Yes/No
Open the kit for disconnecting the patient	Yes/No
Pour 2% chlorhexidine solution into a bowl	Yes/No
Perform hand hygiene and put on sterile gloves	Yes/No
Put gauze pads in the chlorhexidine bowl	Yes/No
Place a sterile towel beneath the catheter	Yes/No
Clean the catheter hubs with the chlorhexidine gauzes	Yes/No
Inject saline and heparin into the catheter	Yes/No
Put sterile caps on the catheter hubs	Yes/No
Put the designated dressing around the catheter hubs	Yes/No
Remove gloves and perform hand hygiene	Yes/No

* Hand hygiene – Hand disinfection using alcohol-based hand rub

**Table 2:** Content of the haemodialysis access handling kit (all material sterile in the kit)

Dressing change	
1 set sterile gloves	
3 clean swabs	
1 empty bowl	
Gauzes	
Tagaderm / Hypodress bandages	
Connecting the patient to the haemodialysis machine (following the dressing change)	
Two 5 ml syringes	
1 set sterile gloves	
1 sterile towel	
1 empty bowl	
Gauzes	
Disconnecting the patient from the haemodialysis machine	
1 empty cup	
1 set sterile gloves	
1 sterile towel	
Gauzes	
1 designated dressing for the catheter hubs	
Tagaderm / Hypodress bandages	
2 sterile caps for the catheter hubs	

### Analysis

Access-related infections per 100 patient months were calculated for each unit, both total and per specific access type. In order to assess changes in infection rates over time we examined whether there was a trend over the years (Mann-Kendall test) and also examined the continuous data in a linear regression model. In order to examine whether access types were associated with infection rates, we generated a linear regression model including access types. All analyses were performed with WINPEPI (Abramson, J.H. WINPEPI updated: computer programs for epidemiologists, and their teaching potential. Epidemiologic Perspectives & Innovations 2011, 8:1).

## Results

**Fig. 1:**
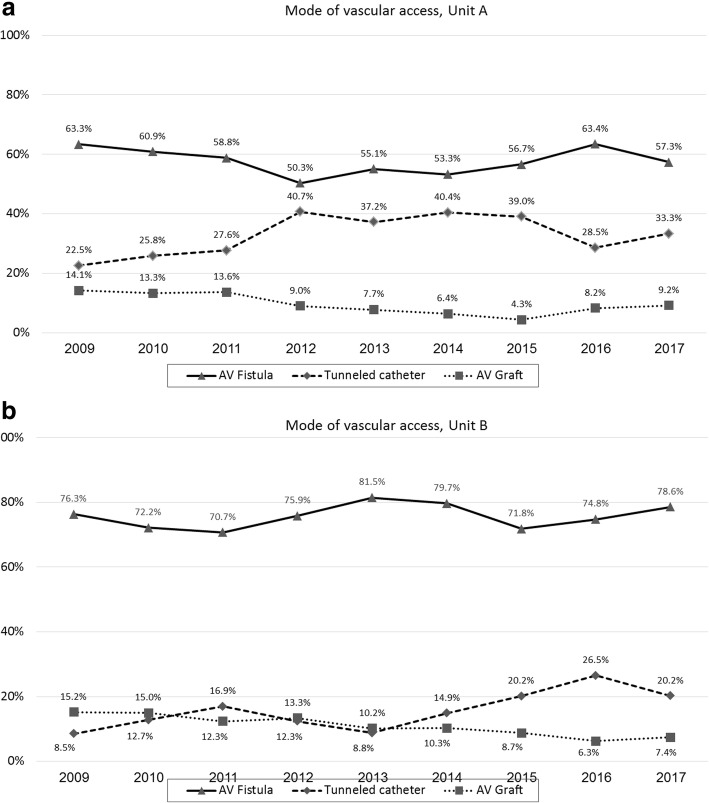
**a, b** The proportions of patients with each mode of vascular access (tunnelled catheter, AV graft or fistula) in Unit A (**a**) and Unit B (**b**) units. Summarized proportions could exceed 100% since some patients had both a tunnelled catheter and an AV fistula or graft in the same time until the fistula or graft were fully operational

During a nine-year period (January 1st 2009 - December 31st 2017), Units A and B treated 4471 and 7547 patients (mean number of patients per year: 497 (range 435–556) and 839 (range 777–1055), respectively). For the majority of patients, the haemodialysis access was through an AV Fistula (66.7%, range 50.3–81.5%, Fig. 1a and b).

While the rate of patients with an AV fistula did not change significantly during the study period, there was a decrease in the rate of graft usage parallel to an increase in tunnelled catheter usage (Fig. 1a, Fig. 1b).

Overall, 938 and 1061 events were reported in the A and B haemodialysis units by the nursing staff during the study period (mean number of events per year: 104 and 118 respectively). The rates of hospitalization, antibiotic usage, bacteremia episodes and access- related infections are shown in Table 3.

**Table 3:** Characteristics of the events* in both dialysis units during a nine-year study period

	Unit A	Unit B
	N (%)	N (%)
Total events	938	1061
Hospitalizations	700 (75)	725 (68)
Treated with antimicrobials	648 (69)	747 (70)
Bacteremia episodes	146 (16)	121 (11)
Access exit site infection^&^	27 (3)	13 (1)
Access-related infections	85 (9)	41 (4)
Pathogens isolated^#^	65 (76)	31 (76)
*Enterobacteriaceae*	30 (46)	8 (26)
*Pseudomonas aeruginosa*	8 (12)	1 (3)
*Staphylococcus aureus*	11 (17)	15 (48)
Other gram positive cocci	13 (20)	6 (19)
Other ^$^	3 (5)	1 (3)

* Events – hospitalization, antibiotic treatment, positive blood culture, vascular exit site infection or

catheter exchange due to infection

^&^ Access exit site infection – tunnelled catheter, 21/27 (78%) in unit A and 11/13 (85%) in unit B; AV graft and AV fistula, each, 3/27 (11%) in unit A and 1/13 (8%) in unit B

^#^ Pathogens could be isolated either from blood culture or exit site infection

^$^ Other – unit A, 2 candida sp. and 1 polymicrobial; unit B, 1 anaerobe

**Fig. 2:**
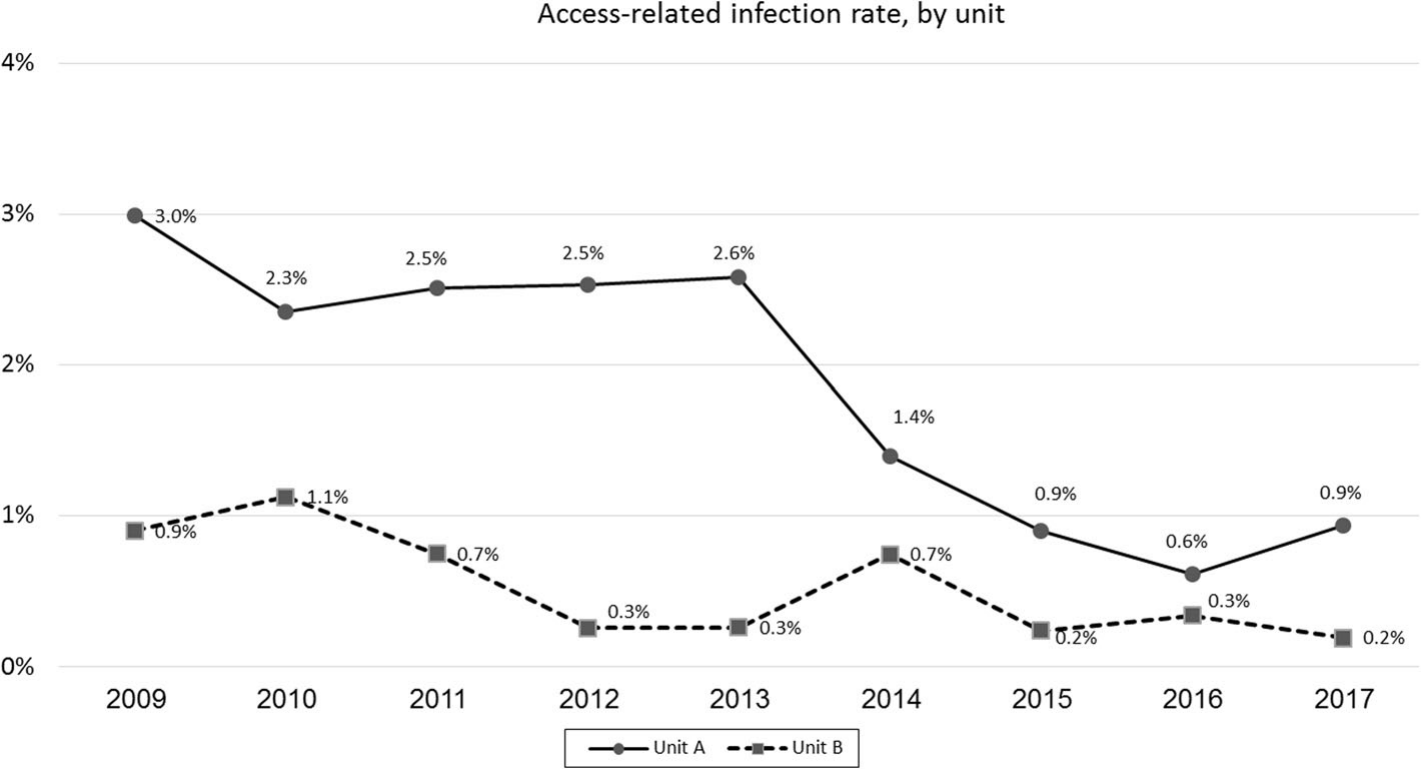
Annually access-related infection rates, by unit. There is a significant trend of decrease in both units (Unit A, from 3.0 to 0.9%, trend: p < 0.05, linear regression: p < 0.001; Unit B, from 0.9 to 0.2%, trend: *p* < 0.05, linear regression: p = 0.01)

**Fig. 3:**
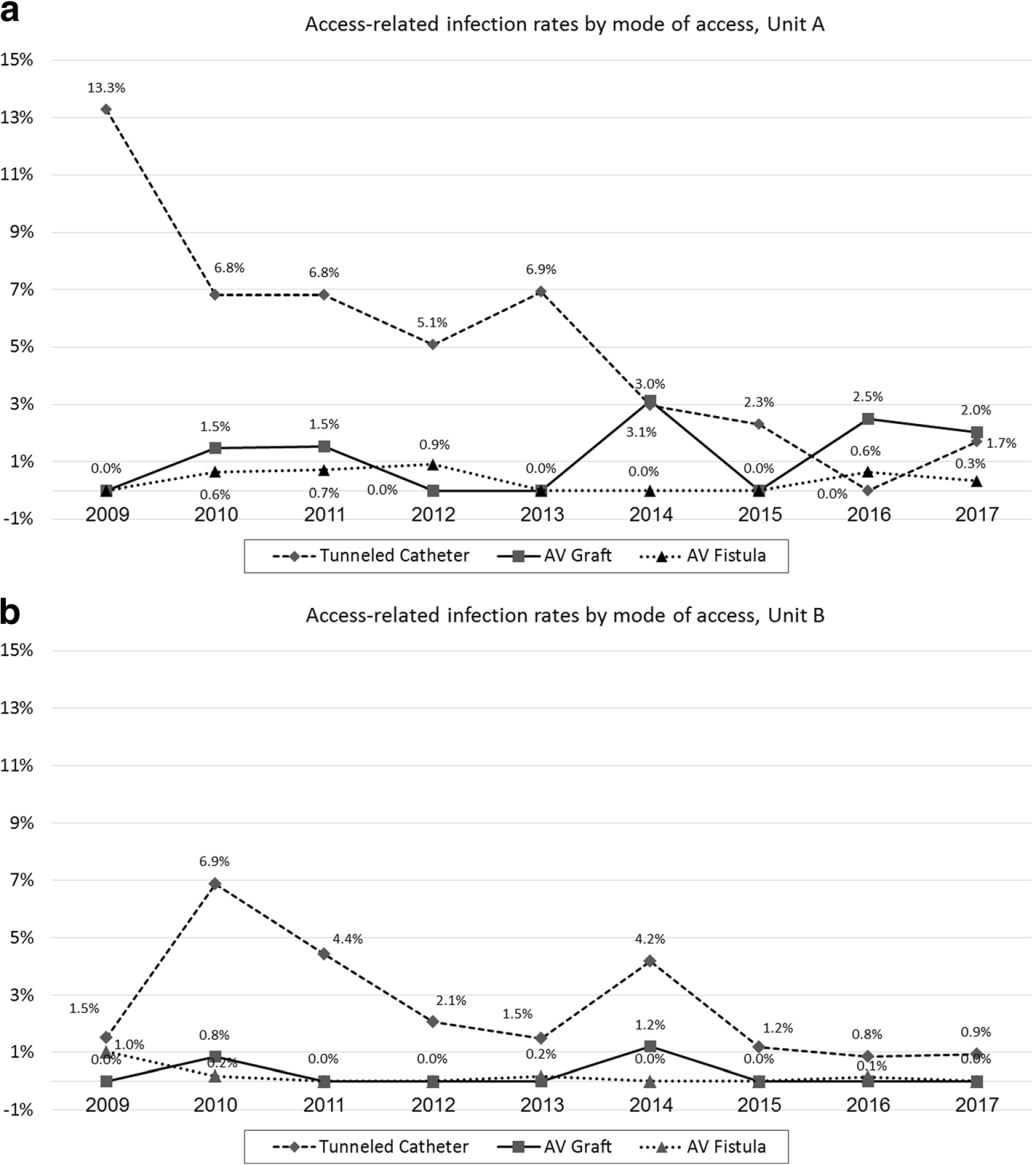
**a, b** Annually access-related infection rates by mode of vascular access (tunnelled catheter, AV graft or fistula) and unit; Unit A (**a**), Unit B (**b**)

During the study period, the access-related infection rate decreased significantly in the two haemodialysis units: from 3 to 0.9% (trend: *p* < 0.05, linear regression: *p* < 0.001) in Unit A and from 0.9 to 0.2% (trend: p < 0.05, linear regression: *p* = 0.01) in Unit B (Fig. 2). The rates of access-related infections were higher in patients using a tunnelled catheter as compared to an AV graft or an AV fistula (Fig. 3a and b, trend: p < 0.05, linear regression: p = 0.01). The isolation of the different pathogens is presented in Table 3. Pathogens were isolated in 65/85 (76.5%) and 31/41 (75.6%) access-related infections in the A and B units respectively. The events in which pathogens were not found represent access-exit site infections.

## Discussion

We demonstrated a significant decrease in the rate of access-related infections in two dialysis units during a nine-year period, by implementing an intervention and surveillance program. The rates decreased even though the usage of tunnelled catheters increased. We believe that the main reason leading to this change was the evaluation of work methods and work space, and following changes in handling the vascular access during the whole dialysis session. Establishing a checklist led to designing a structured kit containing all the equipment needed to perform the procedures, arranged according to the order of steps during the dialysis session. We believe that the implementation of an intervention and surveillance program in which the dialysis units’ nurses were involved, and the resulting increased awareness of the staff to the prevention of access-related infections contributed to the decrease in access-related infections as well. The -related infection rate corresponds with the mean access-related infection rates reported in the National Healthcare Safety Network (NHSN) Dialysis Event Surveillance Report for 2014, as 1.93% [8].

The ability to reduce central line associated bloodstream infection (CLABSI) by implementing a quality and improvement intervention in intensive care units has been demonstrated previously [9]. This approach was also applicable and effective in haemodialysis patients [4, 10]. Our study emphasizes the need for checklists and bundles for handling the vascular access in haemodialysis patients, an approach that has been implemented mostly in intensive care unit patients but is less reported in haemodialysis units [11, 12]. Establishing this checklist also guided us in design and use of a dedicated kit containing all of the necessary equipment for handling the vascular access during the whole haemodialysis session, including dressing of the vascular access. Using a readymade kit reduces the need to gather equipment for the procedure manually and has been shown by Pronovost et-al to decrease CLABSI rates in intensive care units [13]. To our knowledge, this approach has not been applied yet in haemodialysis units.

Another tool for the prevention of catheter related infection is the use of antibiotic lock at the end of each haemodialysis session. Some randomized controlled studies and meta-analyses have been done assessing the role of antibiotic locks for the prevention of access-related infections in haemodialysis patients and showed a reduction in bacteremia rates. However, most had a short duration of follow-up. So long-term benefit or loss of efficacy, development of antimicrobial resistance, or other adverse effects could not be evaluated [14]. Additionally, this practice is not part of the current guidelines [7]. In our institution, we use antibiotic lock as salvage therapy only, in patients with complicated access-related infections.

The decrease in access-related infections demonstrated in our work occurred despite an increase in the proportion of patients using tunnelled central venous catheters. It is known that tunnelled dialysis lines are prone to get infected about 20 times more often than AV fistulas and ten times more often than intravenous grafts [3]. The increased proportion of patients using tunnelled lines in our dialysis units is not consistent with trends of vascular access types in haemodialysis patients [15]. This unestimated effect occurred despite our efforts to increase the proportion of patients using AV fistula or graft. The increase in the proportion of patients using tunnelled lines might be explained by the fact that our units are hospital based, allowing for increased medical supervision, and therefore treat more complicated and elderly patients that are not always candidates for an AV fistula operation.

Our study has several limitations. We did not collect demographic and clinical data about our haemodialysis patient population; hence we could not compare the patients’ characteristics in the 2 units. Even though, the effect of our surveillance and intervention program remains significant and consistent over time in both haemodialysis units. Additionally, we relied on events reporting done by the haemodialysis nursing staff. However, we believe that our surveillance was sensitive enough since the reports included not only bloodstream infections but also fever episodes, antibiotic treatments started and hospitalizations.

## Conclusions

In conclusion, we demonstrated that by implementing an intervention and surveillance program and by using a dedicated checklist and readymade kit for handling the vascular access we were able to significantly lower the access-related infection rates, even in the presence of a high proportion of tunnelled central venous catheters.

## Data Availability

The datasets used and/or analysed during the current study are available from the corresponding author on reasonable request.

## Abbreviations

AV: Arteriovenous
CDC: Centers for Disease Control and Prevention
CLABSI: central line associated bloodstream infection
